# Mentimeter Improves Student Engagement in Online Clinical Anatomy Revision Sessions: A Programme Evaluation

**DOI:** 10.7759/cureus.74178

**Published:** 2024-11-21

**Authors:** Edward J Armstrong, Louise Rogers, Mia Lyon, Joanne L Selway

**Affiliations:** 1 Medical Education, University of Buckingham Medical School, Buckingham, GBR

**Keywords:** active learning, anatomy, audience response systems, engagement, interactivity, online learning

## Abstract

Background

There is a need to develop evidence-based strategies to maximise student engagement in online learning contexts. Audience response systems (ARS) are well-established active learning tools that improve engagement in the face-to-face classroom, but they remain under-researched in the online context.

Aims

This study aimed to directly compare student engagement, perceptions and learning following clinical anatomy revision sessions including interactive questions asked using an ARS (Mentimeter) compared to interactive questions asked using videoconferencing interaction only.

Materials and methods

We designed a series of 11 online clinical anatomy revision sessions advertised to all undergraduate medical students at a UK university. Five of these sessions were randomised to include interactive questions asked using an ARS (Mentimeter), and the remainder included interactive questions asked using videoconferencing (Microsoft Teams) functionality only. Data on attendance and engagement were collected, and attendees were asked to complete an end-of-session survey and an end-of-session test of knowledge.

Results

On average, significantly more attendees engaged with each interactive question asked using Mentimeter compared to questions asked without (30.1% vs 6.4%; p=2.20×10^-16^). Students scored higher on the end-of-session test of knowledge following sessions using Mentimeter (6.6 vs 5.3; p=0.007) and had a higher survey response rate (24.6% vs 14.3%; p=0.003). Students reported that Mentimeter improved engagement, interest, learning and testing of knowledge.

Conclusions

This study adds to the existing literature by demonstrating student engagement is significantly higher when using ARS (Mentimeter) to ask interactive questions during online revision sessions than using videoconferencing interaction alone.

## Introduction

The lockdown and/or restriction of movement during the COVID-19 pandemic resulted in a widespread pivot of higher education activity from campus-based teaching to online education [1,2]. Medical education has traditionally been taught in apprenticeship or social learning models with face-to-face teaching therefore seeming to be a pre-requisite.

Whilst the acute transition of medical education to an online format occurred as an emergency, there had been a pre-existing movement towards online learning in medical education, and online and blended learning is likely to continue to be a prominent component of medical undergraduate curricula going forward [2-5].

Several studies have raised concerns about implementing widespread educational technology changes without adequate research [2,5,6]. Challenges to the successful implementation of online and blended learning in medical education include access to technology, technological issues, faculty and student competence with new online technology and specific issues teaching practical skills [1,7]. Student engagement and a lack of communication between students and teachers, including difficulty assessing social cues, have also been cited as barriers to implementation [2,5,8].

Proposed solutions to issues with online engagement include the use of tools to encourage active learning, such as online polling and breakout room discussion [2,6]. Many of these strategies remain under-researched in the context of online learning, and there is a need to validate the utility of these tools in the online context [2].

Audience response systems (ARS) are now well recognised as pedagogical tools that promote active learning in face-to-face classrooms [9]. Students across multiple disciplines report positive attitudes around the use of ARS and higher engagement when ARS are used during in-person teaching activities [10-13]. ARS have been demonstrated to have a positive objective effect on face-to-face learning in the short term [14], although their impact on longer-term learning is less clear [15-17].

The migration of medical education to online learning during the COVID-19 pandemic prompted commentators to highlight features of ARS particularly suited to this context. These include the flexibility of currently available online ARS, where students are able to answer on their own personal devices without requiring additional software and interactive questions can be amalgamated into PowerPoint presentations [18]. They are thought to scale well for large groups [19] and bridge the physical divide to allow students to become active online participants [20].

Kremer et al. surveyed medical students following the use of an ARS for online diagnostic teaching, with participants reporting a significant increase in student-teacher interaction using ARS and a majority stating a subjectively higher learning effect when compared to conventional online seminars [20]. Another study demonstrated improved short-term knowledge outcomes following ARS-enhanced online histology teaching sessions compared to traditional didactic online teaching [21]. Overall, however, there is a relative paucity of research evaluating the utility of ARS in the online or virtual context. Given the challenges of online learning, investigation of the impact of ARS on objective student engagement in the virtual context would therefore be useful.

This programme evaluation aimed to directly compare online clinical anatomy revision sessions including interactive questions asked using an ARS against those including interactive questions asked without an ARS. The primary aim was to evaluate the impact of ARS on student engagement, learning and perceptions. We hypothesised that ARS use would substantially increase objective student engagement and increase short-term knowledge outcomes. A secondary aim was to identify barriers to student engagement with interactive questions in this educational context.

## Materials and methods

Educational context

The University of Buckingham Medical School (UBMS) is an independent UK medical school offering a condensed 4.5-year undergraduate medical degree (MB ChB). The integrated course is divided into Phase 1 (392 students, 57.4%), based primarily in the classroom, and Phase 2 (291 students, 42.6%), based in the clinical environment. Phase 1 comprises a series of integrated systems units presented in a clinical context. Phase 2 rotations are both system and departmentally structured and situated across primary and secondary care placements [22]. The mean age of current students at UBMS is 22 and 59.6% of students are female.

Mentimeter

Mentimeter (Stockholm, Sweden) is a web-based ARS that allows instructors to ask questions during presentations and attendees to answer on personal electronic devices, including mobile phones and laptops, using a web browser. Mentimeter offers several question types including multiple choice, word cloud, open-ended and ranking questions. Presenters can create presentation slides on the Mentimeter platform, upload Microsoft (MS) PowerPoint slides to the platform or use the Mentimeter PowerPoint plugin [23]. We produced two 10-minute tutorial videos in-house explaining how to create presentations and present using Mentimeter and circulated these to all staff involved in the teaching programme.

Programme design

We planned a series of 11 sessions based on UBMS Phase 2 rotations, each 45-60 minutes long. The learning objectives for the sessions were formulated collaboratively with Phase 2 clinical staff and included up to five pathologies and associated anatomy in each session. Each session was delivered virtually on MS Teams by a different presenter, who were all clinicians involved in Phase 1 teaching.

Educators were instructed to introduce each relevant pathology with an interactive multiple-choice question and encouraged to use interactive questions throughout their session. All educators were sent the in-house Mentimeter tutorial videos and were invited to attend two sessions clarifying the instructions given to participating educators. Five presenters (45.5%) were randomised with a simple randomisation process to use the Mentimeter ARS, and the remaining six (54.5%) were asked to interact with students verbally and using the chat function on MS Teams.

Student attendance was not mandatory and all UBMS students were invited to attend. All sessions were recorded, and the recordings, along with PowerPoint slides, were released to all UBMS students 72 hours after each session.

Evaluation methods: attendance and engagement

Attendance records were extracted from MS Teams with any student joining the MS Teams meeting considered to have attended. The number of students answering interactive questions via the ARS was tallied automatically by the Mentimeter software. The number of verbal and written MS Teams responses was manually collected by watching session recordings and tallying student responses.

Evaluation methods: end-of-session survey

After all sessions, attendees were asked to complete an 11-item survey (excluding administrative questions) developed collaboratively by the authors assessing engagement and perceptions of the session and interactive questions. Three authors (EA, ML, JS) checked the face and content validity of the survey. This comprised six questions answered using a 5-point Likert-style scale, four open-ended questions and one question seeking informed consent (Table 1). Four additional Likert-style questions and one open-ended question were asked following sessions using Mentimeter (Table 2).

**Table 1:** End-of-session survey items asked after all sessions

Question	Answer options
How engaged did you feel in the session?	1=not engaged at all
	2=not engaged
	3=neutral
	4=engaged
	5=very engaged
Did you answer any of the interactive multiple-choice questions (by answering verbally, on the Teams chat or through Mentimeter)?	All of the questions
	Most of the questions
	Some of the questions
	I did not engage with any questions
What factors made you more likely to answer the interactive multiple-choice questions?	Free text
What factors made you less likely to answer the interactive multiple-choice questions?	Free text
How useful was the session overall for your learning?	1=not useful at all
	2=not useful
	3=neutral
	4=useful
	5=very useful
How useful were the interactive questions for testing your knowledge?	1=not useful at all
	2=not useful
	3=neutral
	4=useful
	5=very useful
How useful were the interactive questions for helping you learn?	1=not useful at all
	2=not useful
	3 = neutral
	4 = useful
	5 = very useful
How relevant was the session to you?	1 = not relevant at all
	2=not relevant
	3=neutral
	4=relevant
	5=very relevant
What was good about the session?	Free text
What could be improved?	Free text
Are you happy for us to use your answers anonymously for audit, quality improvement and research purposes?	Yes
	No

**Table 2:** End-of-session survey items asked after sessions using Mentimeter

Question	Answer options
Do you feel the use of Mentimeter made the following better or worse?	Answer options below
Engagement	Much worse
	Worse
	No change
	Better
	Much better
Testing of knowledge	Much worse
	Worse
	No change
	Better
	Much better
Enjoyment	Much worse
	Worse
	No change
	Better
	Much better
Learning	Much worse
	Worse
	No change
	Better
	Much better
Please explain your answers to the questions above	Free text

Student answers to open-ended questions were evaluated using a collaborative thematic analysis approach similar to that described by Naeem et al. [24]. Two authors (EA and LS) independently assigned initial codes to every participant's answer to the open-ended survey questions. Codes were assigned using participants' own language (in vivo codes) where possible, and multiple codes were assigned to each answer where necessary. The coding was then discussed and refined by the authors until a consensus was reached. Initial codes were grouped together into categories, firstly independently, and then refined through collaborative discussion to reach a consensus. Finally, this iterative process was repeated to group categories together into themes.

Evaluation methods: end-of-session test of knowledge

All student attendees were invited to complete a 10-question end-of-session multiple-choice quiz on material from each session. All questions were written by the lead author, and the validity of each question was checked by at least one other author.

Data analysis

Data was analysed using MS Excel and RStudio (R Foundation for Statistical Computing, Vienna, Austria (https://www.R-project.org/)). Data was tested for normality using the Shapiro-Wilk test. Data could not be transformed to achieve normality and so non-parametric tests were used. Statistical significance was determined using Wilcoxon's signed-rank test, the Kruskal-Wallis test and Fisher's exact test.

Ethical approval

Approval was granted by the University of Buckingham Faculty of Medicine and Health Sciences Ethics Committee (approval number: 237237855) for the analysis and publication of retrospectively available attendance, engagement and evaluation data on 26/7/24. This teaching programme was developed as a component of routine teaching activity at the University of Buckingham. End-of-session surveys and end-of-session quizzes were administered as a component of routine internal programme evaluation, audit and quality improvement. Informed consent was obtained from survey participants.

## Results

Attendance records

There was a total of 525 unique attendances across the programme. Two hundred and sixty attendances were at sessions using Mentimeter and 265 were at sessions using MS Teams interactivity only (Table 3). The mean number of attendees for each session was 47.7±7.2 (mean±standard error (SE)). Two hundred and forty attendances were from Phase 1 students and 276 from Phase 2 students.

**Table 3:** Comparison between sessions using Mentimeter and sessions using MS Teams interactivity only Data was analysed using Wilcoxon's signed-rank test. p<0.05 was considered significant. MS: Microsoft; SE: standard error

Domain	Sessions using Mentimeter	Sessions using MS Teams interactivity only
Total attendances	260	265
Mean engagement rate/question (mean±SE)	30.1%±1.0%	6.4%±0.4%
End-of-session survey response rate across the programme	24.6%	14.3%
End-of-session quiz response rate across the programme	19.2%	18.5%
Mean end-of-session quiz score (mean±SE)	6.6±0.3	5.3±0.3

Student engagement with interactive questions

**Figure 1:**
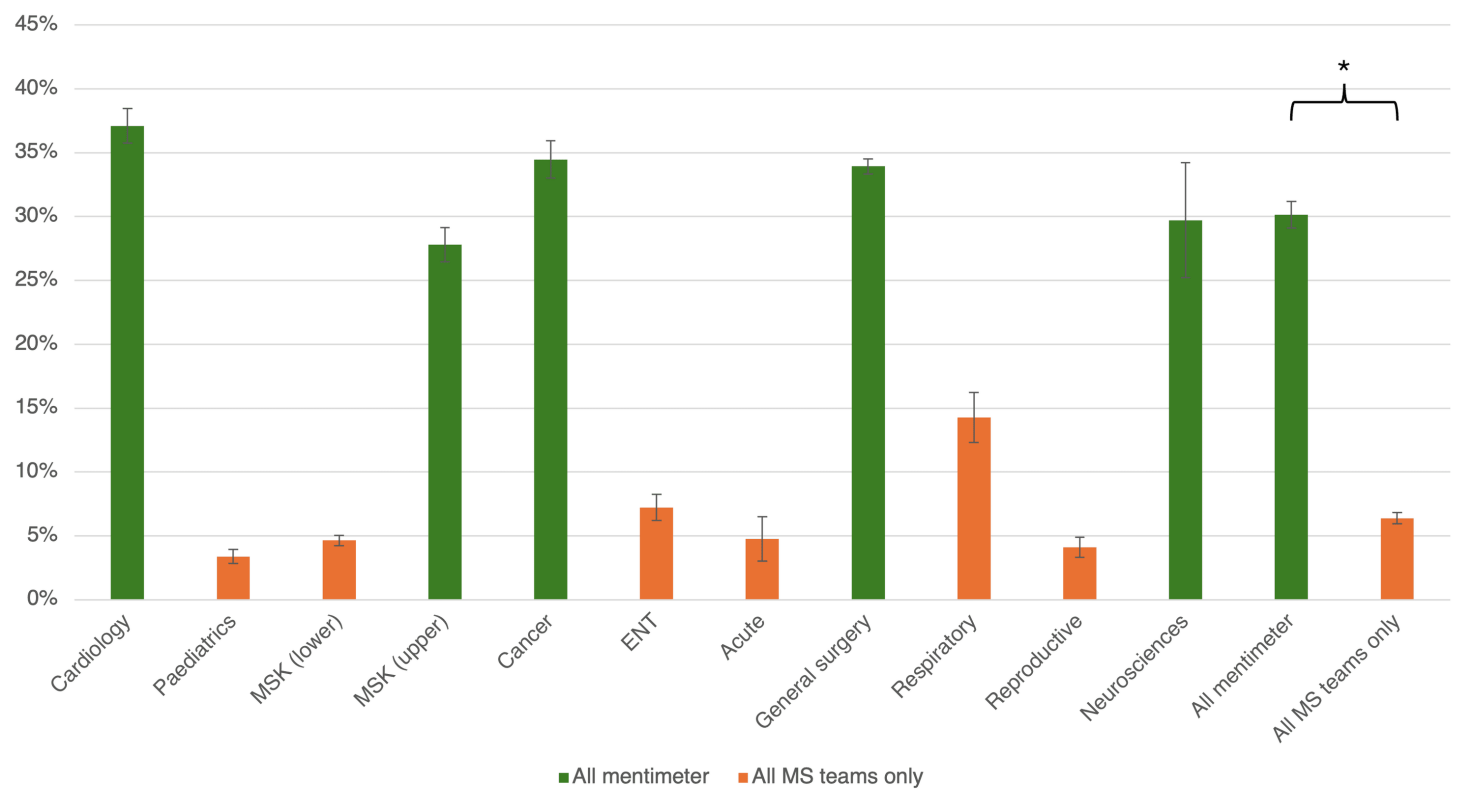
Mean percentage of attendee engagement with each interactive question by session Sessions are ordered chronologically from left to right. * indicates statistical significance. Bars represent standard error. Data was analysed using Wilcoxon's signed-rank test. p<0.05 was considered significant. MSK: musculoskeletal; ENT: ear, nose and throat; MS: Microsoft

The mean number of interactive questions asked in each session was 17.5±2.6. Across all interactive questions asked throughout the programme, the mean engagement rate for questions asked using Mentimeter was 30.1%±1.0%, statistically significantly higher than the mean engagement rate for questions asked using MS Teams interactivity only (6.4%±0.4%) (p=2.20×10^-16^; common language effect size=1.00) (Figure 1).

End-of-session survey results

A total of 102 survey responses were received across the programme, giving an overall feedback response rate of 19.4%. The response rate for sessions using Mentimeter was 24.6% (64 responses), statistically significantly higher than the response rate for sessions using MS Teams interactivity only (14.3%, 38 responses) (p=0.003, Wilcoxon's signed-rank test).

Survey Results: Likert-Style Responses

Students rated both formats highly for engagement, relevance, testing of knowledge and usefulness for learning (Table 4). There were no statistically significant differences between sessions using Mentimeter and sessions using MS Teams interactivity only or between Phase 1 and Phase 2 responders for any questions reported in Table 4.

**Table 4:** Mean responses to survey items asked using a five-point Likert-style scale Data was analysed using the Kruskal-Wallis test. p<0.05 was considered significant.

	1-point option	5-point option	Sessions using Mentimeter (mean score)			Sessions using MS Teams interactivity only (mean score)			P-value
			All	Phase 1	Phase 2	All	Phase 1	Phase 2	
How engaged did you feel in the session?	Not engaged at all (1)	Very engaged (5)	4.63	4.71	4.58	4.54	4.47	4.60	0.449
How useful was the session overall for your learning?	Not useful at all (1)	Very useful (5)	4.78	4.86	4.74	4.87	4.94	4.80	0.815
How useful were the interactive questions for testing your knowledge?	Not useful at all (1)	Very useful (5)	4.75	4.80	4.72	4.76	4.89	4.70	0.794
How useful were the interactive questions for helping you learn?	Not useful at all (1)	Very useful (5)	4.79	4.90	4.77	4.80	4.94	4.65	0.126
How relevant was the session to you?	Not relevant at all (1)	Very relevant (5)	4.76	4.75	4.77	4.70	4.83	4.70	0.967

Around 81.3% of students reported answering "All of" or "Most of" the interactive questions during sessions using Mentimeter compared to 47.4% of students in sessions using MS Teams interactivity only (p=0.0004, Wilcoxon's signed-rank test) (Table 5).

**Table 5:** Responses to survey item: "Did you answer any of the interactive multiple-choice questions?" Data was analysed using Fisher's exact test; p=1.60×10^-5^. p<0.05 was considered significant.

Options		All of the questions	Most of the questions	Some of the questions	I did not engage with any questions
Sessions using Mentimeter (number of answers (percentage))	All	40 (62.5)	12 (18.8)	6 (9.4)	6 (9.4)
	Phase 1	12 (57.1)	4 (19.0)	3 (14.3)	2 (9.5)
	Phase 2	28 (65.1)	8 (18.6)	3 (7.0)	4 (9.3)
Sessions using MS Teams interactivity only (number of answers (percentage))	All	4 (10.5)	14 (36.8)	11 (28.9)	9 (23.7)
	Phase 1	0 (0.0)	7 (38.9)	6 (27.8)	6 (33.3)
	Phase 2	4 (20.0)	7 (35.0)	6 (30.0)	3 (15.0)

Survey Results: ARS-Specific Questions

**Figure 2:**
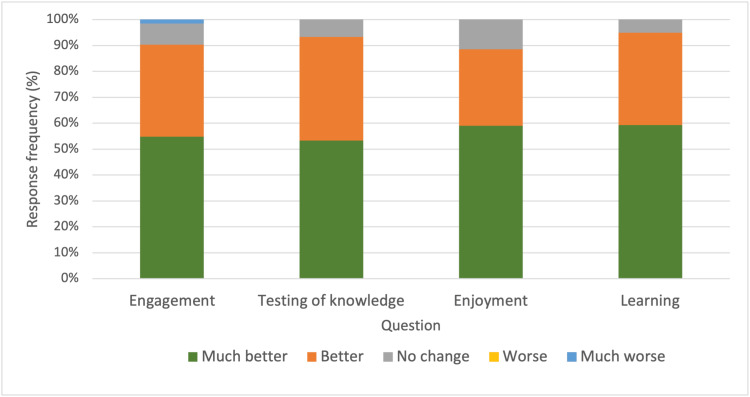
Responses to survey item: "Do you feel the use of Mentimeter made the following better or worse?"

The majority of students reported that Mentimeter made engagement (90.3%), testing of knowledge (93.3%), enjoyment (88.5%) and learning (94.9%) "Much better" or "Better" (Figure 2).

Survey Results: "What Factors Made You More/Less Likely to Answer the Interactive Multiple-Choice Questions?"

We identified three themes amongst student answers to the questions "What factors made you more/less likely to answer the interactive multiple-choice questions?": question properties, the impact of peers and session presentation.

Theme 1: question properties: The most frequently cited factor making students more likely to answer the interactive questions was questions aimed at an appropriate level (referenced by 34 responses). Similarly, questions aimed at an inappropriate level was the most frequently cited factor making students less likely to engage (33 responses). Students also identified the question type (multiple-choice questions/single best answer questions) (seven responses), clarity (six responses) and clinical orientation of the interactive questions (four responses) as positive factors.

Theme 2: the impact of peers: The impact of peers was more likely to be referenced as a positive factor following sessions using Mentimeter and a negative factor following sessions using MS Teams interactivity only. Students suggested the anonymity (nine responses) and opportunity for competition (two responses) afforded by Mentimeter increased their likelihood of engaging with the interactive questions.

Theme 3: session presentation: Some responses cited the ratio of time spent on questions versus explanations as a positive factor. The time provided to answer the interactive questions was identified as both a positive and negative factor for different sessions. Only two students identified device access as a barrier to engagement with interactive questions.

Survey Results: Answers to Other Open-Ended Questions

Open-ended ARS-specific question: Anonymity and interactivity were identified as positive features of Mentimeter when students were asked to explain their answers to the ARS-specific questions in Table 2.

Answers to other questions: When asked "What was good about the session?", students identified similar factors to those described above, also praising the interactivity of the sessions and the exam relevance of the content and questions. The most frequent improvement suggested was a higher volume of teaching, including more questions and longer and more frequent sessions.

End-of-session test of knowledge results

A quiz attempt was considered "valid" if it occurred within 72 hours of the teaching session and included no more than five unanswered questions. The response rate for "valid" quiz attempts was 19%. There was no difference between the response rates following sessions using the ARS (19.2%) and sessions using MS Teams interactivity only (18.5%).

The mean score on quizzes following sessions using Mentimeter was 6.6±0.3 out of 10, compared to 5.3±0.3 for those using MS Teams interactivity only (p=0.007, Wilcoxon's signed-rank test; common language effect size=0.667).

## Discussion

We report an evaluation of a clinical anatomy online revision programme taught to an undergraduate medical student group of mixed levels. The key novel finding of this report was substantially greater objective student engagement with interactive questions asked using the ARS compared to videoconferencing (MS Teams) functionality only (30.1% engagement/question vs 6.4%; p=2.20×10^-16^). When asked directly, students reported that Mentimeter improved engagement, testing of knowledge, enjoyment and learning. This is broadly consistent with the findings of other studies describing ARS use for in-person teaching [11-14,20,25]. Students scored higher on the end-of-session test of knowledge following sessions using Mentimeter (6.6 vs 5.3; p=0.007) and had a higher survey response rate (24.6% vs 14.3%; p=0.003).

Castelli and Sarvary described difficulties engaging students on videoconferencing software, including many students choosing to switch their cameras off [6], which makes it difficult for educators to assess social cues effectively [2]. Interestingly, no participants switched their video cameras on during the programme evaluated in the current study, although this was neither mandated nor encouraged. Castelli and Sarvary emphasise the importance of active learning techniques to engage students and finding alternative means of interaction beyond verbal and non-verbal communication via webcams [6]. Active learning was also identified by Koh and Daniel as a major student engagement strategy used for online education during the COVID-19 pandemic. They highlight the importance of pedagogical innovation to fully leverage the opportunities afforded by the virtual setting [2]. Our findings support ARS as an evidence-based tool for improving objective student engagement in online sessions, which are likely to be a prominent component of medical undergraduate curricula going forward [2-5].

The student cohort participating in this programme was of mixed experience. We did not identify a statistically significant difference in the perceptions of students at different levels of learning, suggesting that ARS are useful for teaching mixed-ability cohorts in online settings. This is consistent with the results of previous studies demonstrating positive perceptions of ARS amongst mixed groups of pre-clinical and clinical students [20] and post-graduate clinicians of different levels [26] during classroom-based teaching.

The factor most frequently cited by students as influencing the likelihood of interaction was questions aimed at an "appropriate level". This contrasts somewhat with previous studies which have identified technology-related issues as key barriers to ARS implementation [10,14,27]. In this study, only two students reported device access as a barrier. One explanation for this is the difficulty in writing interactive questions at an appropriate level for all students in our mixed-ability cohort. Another factor is differential access to a secure internet connection; this is likely to be better amongst the participants in this programme than internet connections in the developing world or rural environments [1,8].

Our survey findings support previous work identifying anonymity and competition as positive aspects of ARS [10,11,14,28]. Interestingly, participants in this study were more likely to cite peer interactions in MS Teams sessions as a factor making them less likely to engage with interactive questions, specifically other students answering quickly. Koh and Daniel, in a systematic review of online education during the COVID-19 pandemic, describe similar issues with online classes eventually becoming "dominated by more vocal students, making the quieter ones left out" [2]. ARS systems such as Mentimeter allow quick responses, but the visualisation of answers is controlled by the educator, so the influence of peer response timing becomes a negligible factor. Our findings provide evidence for ARS in enabling the participation of a broader cross section of students, not just those "more vocal" students.

Each session in this programme was delivered by a different educator. Beyond instructions on the use of interactive questions, this study did not control explicitly for presentation style and so this is a potential confounding factor. Similarly, content may have varied in difficulty with topic between sessions. On the other hand, this study demonstrates consistently higher objective student engagement across a sample of 11 educators, providing some evidence that ARS are compatible with, and enhance, a diversity of different teaching styles. We did not investigate the perceptions or experiences of educators in this study, and this is an avenue for further work.

The salient limitation of this study is the low end-of-session survey and test of knowledge response rates, which affect the reliability of the reported quantitative survey and quiz data. The end-of-session test of knowledge assessed short-term knowledge retention only; further work could explore the longer-term knowledge outcomes of ARS use in online teaching, which have been mixed in the face-to-face setting [15-17]. The population studied represents a self-selecting group of voluntary attendees in a single institution and so may not be fully representative of the general population of UK medical students. Further work could replicate this study in the context of mandatory teaching and expand on the limited qualitative data we have reported here, recognising the diversity of student experiences influencing engagement.

## Conclusions

This study suggests that ARS are useful evidence-based tools for engaging students with interactive questions in online revision sessions. They are perceived positively by students and have a positive impact on short-term knowledge retention. ARS may be particularly useful for classes of mixed ability and may mitigate negative attributes of peer influence in online learning.
